# Weight-Related Negative Experiences, Internalized Weight Stigma, and General Self-Efficacy in Adults with Obesity: An Explanatory Sequential Mixed-Methods Study

**DOI:** 10.3390/healthcare14203510

**Published:** 2026-10-19

**Authors:** Bilgehan Açıkgöz, Ayşegül Ekemen, Meltem Cemre Meydan, Taner Bayraktaroğlu, Ferruh Niyazi Ayoğlu

**Affiliations:** 1 Department of Public Health, Faculty of Medicine, Zonguldak Bülent Ecevit University, 67600 Zonguldak, Türkiye; bilgehanacikgoz@yahoo.com (B.A.); mcmeydan15@gmail.com (M.C.M.); fayoglu@yahoo.com (F.N.A.); 2 Division of Endocrinology and Metabolism, Department of Internal Medicine, Faculty of Medicine, Zonguldak Bülent Ecevit University, 67600 Zonguldak, Türkiye; baytaner@yahoo.com

**Keywords:** obesity, weight stigma, internalized weight stigma, self-efficacy, healthcare experiences, patient-centered care, mixed methods

## Abstract

**Highlights:**

**What are the main findings?**

• Weight-related negative experiences were associated with greater internalized weight stigma and lower general self-efficacy, with a significant indirect association through internalized stigma.
• Among participants reporting weight-related negative healthcare experiences, most follow-up respondents reported an effect on subsequent healthcare use.

**What are the implications of the main findings?**

• Qualitative accounts indicate that stigma-informed obesity care should address internalized stigma alongside interpersonal experiences of care.
• Patient-centered communication and size-inclusive healthcare environments may help reduce barriers to healthcare engagement.

**Abstract:**

**Background/Objectives:** Weight stigma may be experienced externally and internalized as self-directed negative beliefs, with consequences for psychological resources and healthcare use. This study examined associations among weight-related negative experiences, internalized weight stigma, and general self-efficacy, and explored how these relationships were reflected in adults receiving obesity care. **Methods:** An explanatory sequential mixed-methods study was conducted at a university-affiliated multidisciplinary obesity and diabetes center in Türkiye. In the quantitative phase, 435 adults with obesity were consecutively recruited during the predefined study period and completed researcher-developed items on weight-related experiences, the Modified Weight Bias Internalization Scale, and the General Self-Efficacy Scale. Multivariable linear regression and bootstrap-based indirect-effect analysis were performed. Subsequently, 20 participants underwent semi-structured interviews and thematic analysis. Findings were integrated using a joint display. **Results:** Weight-related discrimination or negative experiences in social or workplace settings were reported by 27.8% of participants, while 11.5% reported weight-related negative healthcare experiences. Of those reporting negative healthcare experiences and responding to the follow-up item, 77.1% stated that subsequent healthcare use had been affected. Social/workplace negative experiences were associated with higher internalized weight stigma scores (B = 0.814, 95% CI 0.525–1.103) and lower general self-efficacy scores (B = −4.238, 95% CI −6.925 to −1.551). The hypothesized indirect association through internalized weight stigma was significant (B = −2.403, 95% CI −3.569 to −1.353). Qualitative themes addressed obesity as label and identity, judgment and internalization, weight overshadowing care, and conditional confidence. **Conclusions:** Weight-related negative experiences, internalized weight stigma, and lower general self-efficacy were interrelated in this treatment-seeking population. Qualitative findings highlighted interpersonal and structural barriers to care.

## 1. Introduction

Obesity is a chronic, multifactorial disease whose prevalence has increased substantially worldwide [1]. In Türkiye, self-reported national survey data indicate an obesity prevalence of 20.2% among people aged 15 years and older [2]. Beyond its biomedical burden, people living with obesity may experience weight stigma: social devaluation based on body weight, expressed through stereotypes, prejudice, and discrimination and often reinforced by narratives of personal responsibility [3,4]. Such experiences can occur in social, occupational, and healthcare settings [4,5]. In healthcare, stigma may contribute to reduced trust, avoidance or delay of care, poorer care experiences, and structural barriers that limit equitable care [5,6,7].

Internalized weight stigma occurs when negative weight-related stereotypes are adopted and applied to the self. It has been associated with poorer psychosocial, physical, and behavioral health, including depression, anxiety, poorer health-related quality of life, higher body mass index (BMI), and maladaptive eating-related outcomes [6]. Importantly, internalized stigma may represent one pathway through which experienced or perceived stigma relates to broader biopsychosocial outcomes [7]. Distinguishing external experiences from their internalization is therefore important when examining how stigma relates to psychological resources and healthcare behavior. These constructs should therefore be examined separately rather than treated as interchangeable aspects of stigma.

The relevance of weight stigma also extends to clinical populations with obesity-related metabolic conditions. In particular, the coexistence of obesity and type 2 diabetes, often termed “diabesity,” represents a context in which internalized weight stigma may be associated with health-related behaviors. In a cross-sectional study of 310 adults with obesity and type 2 diabetes attending primary care, Wong et al. found that higher internalized weight stigma was associated with lower odds of achieving adequate physical activity [8]. Beyond metabolic outcomes and health behaviors, qualitative evidence highlights the importance of patients’ experiences within healthcare settings: Robinson et al. identified accounts of lack of help and empathy, exposure and embarrassment, and weight-focused encounters, alongside positive healthcare experiences [9]. These findings complement previous qualitative evidence syntheses documenting the interpersonal, psychosocial, and structural dimensions of living with obesity and experiencing weight stigma in healthcare [10,11] and are directly relevant to multidisciplinary settings in which obesity and diabetes care are delivered together, as in the present study.

General self-efficacy refers to broad beliefs about one’s capacity to manage demanding situations and differs from domain-specific self-efficacy tied to particular behaviors [12]. As a domain-independent personal resource, general self-efficacy may shape how individuals appraise and respond to adversity beyond weight-management contexts. Multinational evidence has linked internalized weight stigma with healthcare avoidance, lower perceived quality of care, and perceived judgment from health professionals [13], raising the question of whether its correlates also extend to broader beliefs about coping and competence.

Several gaps remain. One previous model in bariatric surgery candidates examined general self-efficacy as an antecedent of weight-bias internalization, with internalization mediating associations with physical activity [12]. By contrast, a recent French clinical study found that internalized weight bias was associated with lower obesity-specific self-efficacy, with coping strategies fully accounting for this association [14]. Whether externally experienced weight-related discrimination or negative experiences are associated with broader, domain-independent self-efficacy remains unclear. In addition, few studies have integrated quantitative modeling of these associations with lived-experience accounts from the same treatment-seeking population; qualitative accounts can add context that statistical associations alone cannot provide, particularly regarding how stigmatizing encounters are interpreted and carried forward into subsequent behavior [11]. Addressing these gaps may clarify whether associations observed within weight-management domains extend to more generalized beliefs about coping and competence.

Accordingly, this mixed-methods study examined internalized weight stigma, general self-efficacy, and healthcare experiences among adults consecutively recruited from a public multidisciplinary obesity and diabetes center. We evaluated the hypothesized indirect association between self-reported weight-related discrimination or negative experiences and general self-efficacy through internalized weight stigma. Weight-related negative healthcare experiences and their reported consequences for service use were examined alongside this model, and qualitative interviews were used to elaborate on how participants described stigma, healthcare encounters, and self-efficacy within the same care setting.

## 2. Materials and Methods

### 2.1. Study Design and Setting

This study employed an explanatory sequential mixed-methods design (QUAN → QUAL). Quantitative data collection was completed before the qualitative phase. Integration occurred during interpretation through a joint display that brought the quantitative and qualitative findings together. The study was conducted at a university-affiliated multidisciplinary obesity and diabetes center in Türkiye. The center functions as a specialized public referral unit providing comprehensive care for obesity and related metabolic conditions at provincial and regional levels. Its outpatient services cover obesity and diabetes care within the same multidisciplinary structure. Given the limited number of Ministry of Health-authorized obesity centers in Türkiye [15], this setting enabled evaluation of a clinically distinct, treatment-seeking population.

### 2.2. Participants and Eligibility Criteria

**Figure 1:**
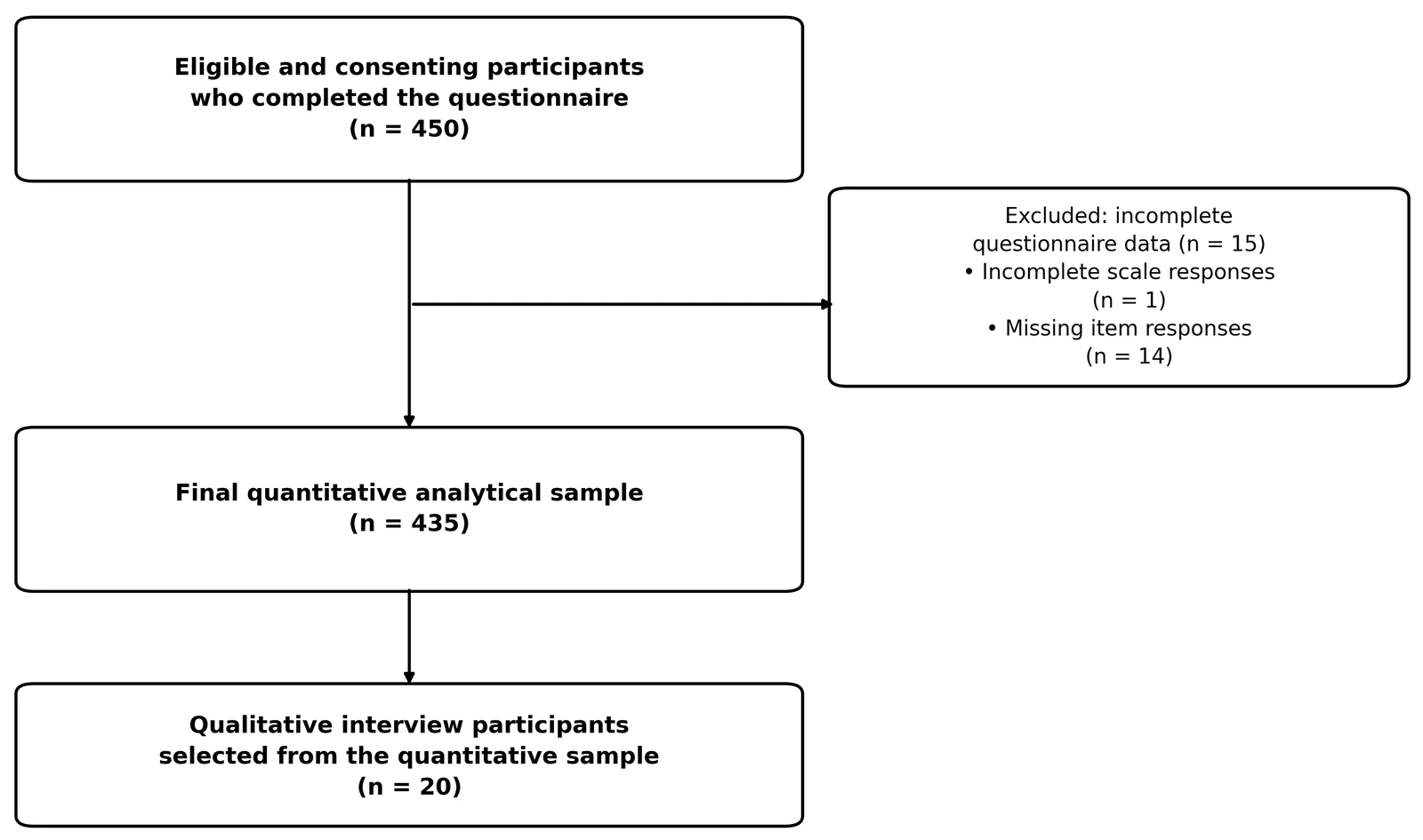
Participant flow diagram. Of 450 eligible and consenting individuals who completed the study questionnaire, 15 records were excluded after data quality checks; the final quantitative analytical sample comprised 435 participants, of whom 20 subsequently completed semi-structured interviews in the qualitative phase. The total number of patients attending the center and the number assessed for eligibility were not recorded.

In the quantitative phase, adults attending the obesity and diabetes outpatient services of the center between February and March 2025 were assessed for eligibility. Inclusion criteria were age ≥ 18 years and BMI ≥ 30 kg/m^2^. The age threshold of ≥18 years was consistent with the WHO definition of the adult population used for overweight and obesity reporting [1]. BMI was calculated from self-reported height and weight. Individuals with cognitive impairment or inability to complete the questionnaire were excluded. Eligible individuals were approached consecutively during the recruitment period and invited to participate. Participation was voluntary, and only those who provided informed consent were enrolled. A total of 450 eligible and consenting individuals completed the study questionnaire. Following data quality checks, 15 questionnaire records were excluded because of incomplete questionnaire data: one with incomplete scale responses and 14 with missing item responses identified during verification against the original questionnaires. The final quantitative analytical sample comprised 435 adults with obesity (Figure 1). The total number of patients attending the center during the recruitment period, the number assessed for eligibility, and the number of eligible individuals who declined participation were not systematically recorded; a participation rate therefore could not be calculated. Obesity was classified according to standard adult BMI categories as Class I (30.0–34.9 kg/m^2^), Class II (35.0–39.9 kg/m^2^), and Class III (≥40.0 kg/m^2^) [16].

In the qualitative phase, 20 participants were recruited from individuals who had completed the quantitative questionnaire during February–March 2025 and subsequently agreed to participate in an interview. The achieved sample included participants of both sexes and across all three obesity classes, with variation in age and in Modified Weight Bias Internalization Scale (MWBIS) and General Self-Efficacy Scale (GSE) scores. These participants were already included in the quantitative analytical sample, and no additional quantitative questionnaires were administered during the qualitative phase. The interviewer did not review participants’ item-level questionnaire responses, and individual scale scores were not discussed during the interviews. Interviews were conducted in April 2025. No formal assessment of thematic saturation was documented during recruitment. The number and characteristics of individuals who declined participation in the qualitative phase were not systematically recorded. Characteristics of the qualitative sample are summarized in Supplementary Table S4.

### 2.3. Variables and Measurements

#### 2.3.1. Researcher-Developed Items

Participants completed 28 researcher-developed items assessing sociodemographic and health characteristics, weight-related experiences, healthcare use, and perceived consequences, informed by previous literature [7,11,13,17]. Two binary exposure variables were used: self-reported weight-related discrimination or negative experiences in workplace or social settings (item 17) and weight-related negative experiences in healthcare (item 24). Because item 17 encompassed both discrimination and broader negative treatment, it was interpreted as a composite indicator rather than discrimination alone. The two exposure domains were analyzed separately and were not combined into a stigma score. The reported effect on subsequent healthcare use was recorded on a four-point scale and dichotomized as affected versus not affected for descriptive analyses. Other researcher-developed items were analyzed descriptively and were not included in the multivariable or indirect-effect models. These items were not intended to constitute a psychometric scale and were not formally pilot or cognitively tested. The full structured questionnaire is provided in Supplementary Material S1. The item on perceived effects of weight on working life was administered to all participants regardless of current employment status and referred to working life in general.

Questionnaires were administered face-to-face during routine clinical visits. Completed forms were reviewed for completeness before data entry, and the dataset was checked for range and logical consistency before analysis. No financial compensation was provided.

The primary outcomes were MWBIS and GSE scores. The explanatory variables were the two binary weight-related exposure variables described above. Covariates in the multivariable models were age, sex, BMI, and perceived income level; the very-low and low perceived-income categories were combined for these analyses. Reported monthly household income in Turkish lira was analyzed separately and used only in bivariate correlations.

#### 2.3.2. Modified Weight Bias Internalization Scale (MWBIS)

Internalized weight stigma was operationalized as internalized weight bias measured by the Modified Weight Bias Internalization Scale (MWBIS). The MWBIS consists of 11 items rated on a 7-point Likert scale, with scores calculated as the mean of all items; higher scores indicate greater internalized weight stigma [18]. The Turkish adaptation reported adequate validity and reliability, with Cronbach’s alpha of 0.92 [19]. In the present sample, internal consistency was good (Cronbach’s alpha = 0.86).

#### 2.3.3. General Self-Efficacy Scale (GSE)

The General Self-Efficacy Scale (GSE) evaluates perceived ability to cope with stressful situations. The scale comprises 17 items rated on a 5-point Likert scale, yielding a total score ranging from 17 to 85; higher scores indicate greater self-efficacy [20]. The Turkish validation study reported Cronbach’s alpha of 0.80 and confirmed the psychometric properties of the scale [21]. Negatively worded items were reverse-scored before summation. In the present sample, internal consistency was good (Cronbach’s alpha = 0.83).

### 2.4. Qualitative Data Collection and Analysis

The semi-structured interview guide was developed a priori from the study objectives and relevant literature on internalized weight stigma, self-efficacy, and healthcare experiences [7,11,13]. It was not individualized according to participants’ quantitative responses. The explanatory linkage between phases was achieved through the sequential conduct of the two phases within the same clinical population and the integration of the two datasets during interpretation using a joint display. The guide covered five domains: obesity perceptions, healthcare encounters, weight management and self-efficacy, psychosocial internalization processes, and policy perspectives. Probing questions were used to explore participants’ experiences and perceived systemic barriers in greater depth. The guide was not formally pilot-tested before data collection.

Interviews were conducted by a female physician researcher holding a medical degree and a specialty in public health, with prior training and experience in qualitative research, who was not involved in the participants’ clinical care. Interviewer and participants had no prior relationship and met for the first time at the interview. Interviews took place face-to-face in a quiet private room at the study site, with no third party present, and lasted approximately 40–45 min each. Interviews were audio-recorded and transcribed verbatim. The semi-structured interview guide is provided as Supplementary Material S2.

Qualitative data were analyzed using a primarily inductive, semantic thematic approach informed by Braun and Clarke [22]. Two researchers independently coded the transcripts and developed an initial codebook. Codes were compared and refined through discussion and then organized into subthemes and overarching themes. The developing thematic structure was reviewed by a third researcher, and disagreements were resolved through consensus. MAXQDA Analytics Pro 2024 (VERBI Software, Berlin, Germany) was used to support data organization and coding. Trustworthiness was enhanced through researcher triangulation and consensus discussions.

### 2.5. Bias

Potential social-desirability bias associated with face-to-face administration was addressed by emphasizing confidentiality. Consecutive recruitment of eligible patients and researcher triangulation in the qualitative analysis were used to limit selection and single-researcher interpretive bias, respectively; however, because the recruitment flow was not fully documented, residual selection bias cannot be excluded.

### 2.6. Study Size and Sample Size Rationale

The quantitative sample comprised 435 eligible adults recruited consecutively during the predefined study period (February–March 2025). No formal a priori sample-size calculation was performed. To characterize the statistical sensitivity of the achieved sample, the minimum detectable correlation was calculated for the achieved sample. With a sample size of 435, a two-sided significance level of 0.05, and 80% statistical power, the minimum detectable correlation was r = 0.134 (Fisher z transformation). The observed correlation between internalized weight stigma and general self-efficacy (ρ = −0.35) exceeded this threshold. This analysis was intended to describe the sensitivity of the achieved sample for detecting a bivariate association and does not establish adequate statistical power for all analyses reported in this study. The qualitative phase comprised 20 semi-structured interviews intended to explore participants’ experiences of weight-related stigma and to provide contextual explanations for the quantitative findings. The adequacy of the qualitative sample was considered in relation to the information power framework proposed by Malterud et al. [23], taking into account the focused research objectives, the specificity and diversity of the experiences represented, and the depth of the interview material; a sample of 20 interviews also exceeds the thresholds at which thematic saturation has typically been reported in empirical interview studies. However, no formal information-power assessment was applied prospectively to determine the sample size, and thematic saturation was not systematically evaluated during recruitment.

### 2.7. Statistical Analysis

Statistical analyses were conducted using IBM SPSS Statistics version 25.0 (IBM Corp., Armonk, NY, USA). Normality was assessed using the Kolmogorov–Smirnov test, histogram inspection, and skewness-kurtosis values. Because continuous variables were not normally distributed, non-parametric tests were applied for group comparisons. Continuous variables are presented as mean ± standard deviation and median (interquartile range), and categorical variables as frequencies and percentages. Categorical variables were compared using the Pearson chi-square test; the Fisher–Freeman–Halton exact test was applied when expected cell counts were below five. Group comparisons of continuous variables were performed using the Mann–Whitney U and Kruskal–Wallis tests, with Dunn–Bonferroni post hoc comparisons following significant omnibus tests. Associations between scale scores were examined using Spearman’s rank correlation coefficient. A two-sided *p* < 0.05 was considered statistically significant.

Multivariable linear regression analyses were conducted to examine covariate-adjusted associations with MWBIS and GSE scores. Age, sex, BMI, perceived income level, self-reported weight-related discrimination or negative experiences, and self-reported weight-related negative experiences in healthcare were entered simultaneously. Model covariates were selected based on the study objectives, theoretical relevance, and previous literature on weight stigma, internalization, and healthcare experiences [7,11,13,17]. The middle perceived-income category served as the reference. Model assumptions were evaluated by inspection of residual plots, assessment of linearity and homoscedasticity, and evaluation of multicollinearity using variance inflation factors (VIF < 5). Unstandardized regression coefficients (B) with 95% confidence intervals (CIs) were reported. Missing data were limited. The multivariable models used complete cases: ten participants (2.3%) with missing data on at least one model variable were excluded, and both models were therefore based on 425 of 435 participants (97.7%). Denominators for individual items varied slightly because of item-level non-response and are reported alongside each proportion. Descriptive analyses were based on available data, and no imputation was performed.

A bootstrap-based indirect-effect analysis was performed using Hayes’ PROCESS macro (Model 4) with 5000 resamples, specifying weight-related discrimination or negative experiences as the exposure, MWBIS as the proposed intermediary variable, and GSE as the outcome. The primary model adjusted for age, sex, and BMI. A sensitivity analysis repeated the model using the full covariate set from the multivariable regressions. An indirect effect was considered statistically significant when its 95% bootstrap CI excluded zero. Given the cross-sectional design, indirect effects were interpreted as hypothesized statistical associations rather than evidence of causal mediation.

### 2.8. Ethics Approval and Consent

Ethical approval was obtained from the Zonguldak Bülent Ecevit University Non-Interventional Clinical Research Ethics Committee, Zonguldak, Türkiye (date: 24 January 2025; Decision No: 2025/03). All participants were informed about the study procedures. In the quantitative phase, participation was voluntary and completion of the questionnaire was considered to indicate consent; in the qualitative phase, written informed consent was obtained from each participant before the interview. This study was reported in accordance with the STROBE and COREQ guidelines; the completed checklists are provided as Supplementary Materials S3 and S4, respectively [24,25].

## 3. Results

### 3.1. Participant Characteristics

A total of 435 adults with obesity were included (mean age 47.3 ± 14.9 years; mean BMI 37.6 ± 6.1 kg/m^2^). Obesity classes I, II and III comprised 40.2%, 31.0% and 28.7% of the sample, respectively; 74.0% were women (Table 1). Qualitative participant characteristics are presented in Supplementary Table S4.

### 3.2. Weight-Related Discrimination and Negative Experiences

#### 3.2.1. Social and Workplace Settings

Overall, 27.8% (120/432) of participants reported weight-related discrimination or negative experiences in workplace or social settings. The proportion reporting these experiences varied by sex, marital status, education, employment status and obesity class, reaching 48.4% in Class III obesity and 66.7% among students; perceived income was not associated (Table 1).

Additionally, among participants who provided a valid response to this item, 44.5% reported that obesity moderately or severely affected their working life; this proportion was higher among those reporting weight-related discrimination or negative experiences (62.2% vs. 37.6%, *p* < 0.001).

Reported psychosocial effects included reduced self-esteem (31.5%), decreased confidence (30.8%), impaired social relationships (27.4%) and hesitation in seeking healthcare (15.6%); 45.8% reported none. Percentages are based on participants providing a valid response to this item, and multiple responses were permitted.

#### 3.2.2. Healthcare Settings

Overall, 11.5% (50/434) of participants reported weight-related negative experiences in healthcare settings, increasing from 5.1% in Class I to 16.8% in Class III obesity (*p* = 0.003). Among those reporting such experiences and responding to the follow-up item, 77.1% (37/48) reported an effect on subsequent healthcare use. Participants reporting healthcare-related negative experiences had higher MWBIS scores (*p* = 0.043) and higher BMI (39.24 vs. 35.97; *p* = 0.002), whereas GSE scores did not differ (*p* = 0.726; Table 2).

Among the six participants whose healthcare use was strongly affected, median MWBIS and GSE scores were 5.64 and 43.00, respectively; because of the very small subgroup size, these values are reported descriptively and no inferential testing is presented for them.

### 3.3. Internalized Weight Stigma and General Self-Efficacy

Median MWBIS and GSE scores were 3.36 (IQR 2.09) and 65.00 (IQR 16.00), respectively. MWBIS was higher among women and participants with Class III obesity, whereas GSE did not differ by obesity class. Participants reporting weight-related discrimination or negative experiences had higher MWBIS and lower GSE scores (both *p* < 0.001; Table 2). MWBIS and GSE were inversely correlated (ρ = −0.35, *p* < 0.001). In a sensitivity analysis excluding the two participants aged 18 years, this correlation was essentially unchanged (ρ = −0.34, *p* < 0.001).

Scores across the remaining sociodemographic characteristics are presented in Supplementary Table S1, and correlations with BMI, age, and household monthly income are presented in Supplementary Table S2.

### 3.4. Multivariable and Indirect-Effect Analyses

In multivariable analysis, weight-related discrimination or negative experiences (B = 0.814, 95% CI 0.525 to 1.103), higher BMI and female sex were independently associated with higher MWBIS scores; the model explained 17.2% of the variance. For GSE, weight-related discrimination or negative experiences was the only variable independently associated with GSE (B = −4.238, 95% CI −6.925 to −1.551; *p* = 0.002), with R^2^ = 0.041 (Table 3).

**Figure 2:**
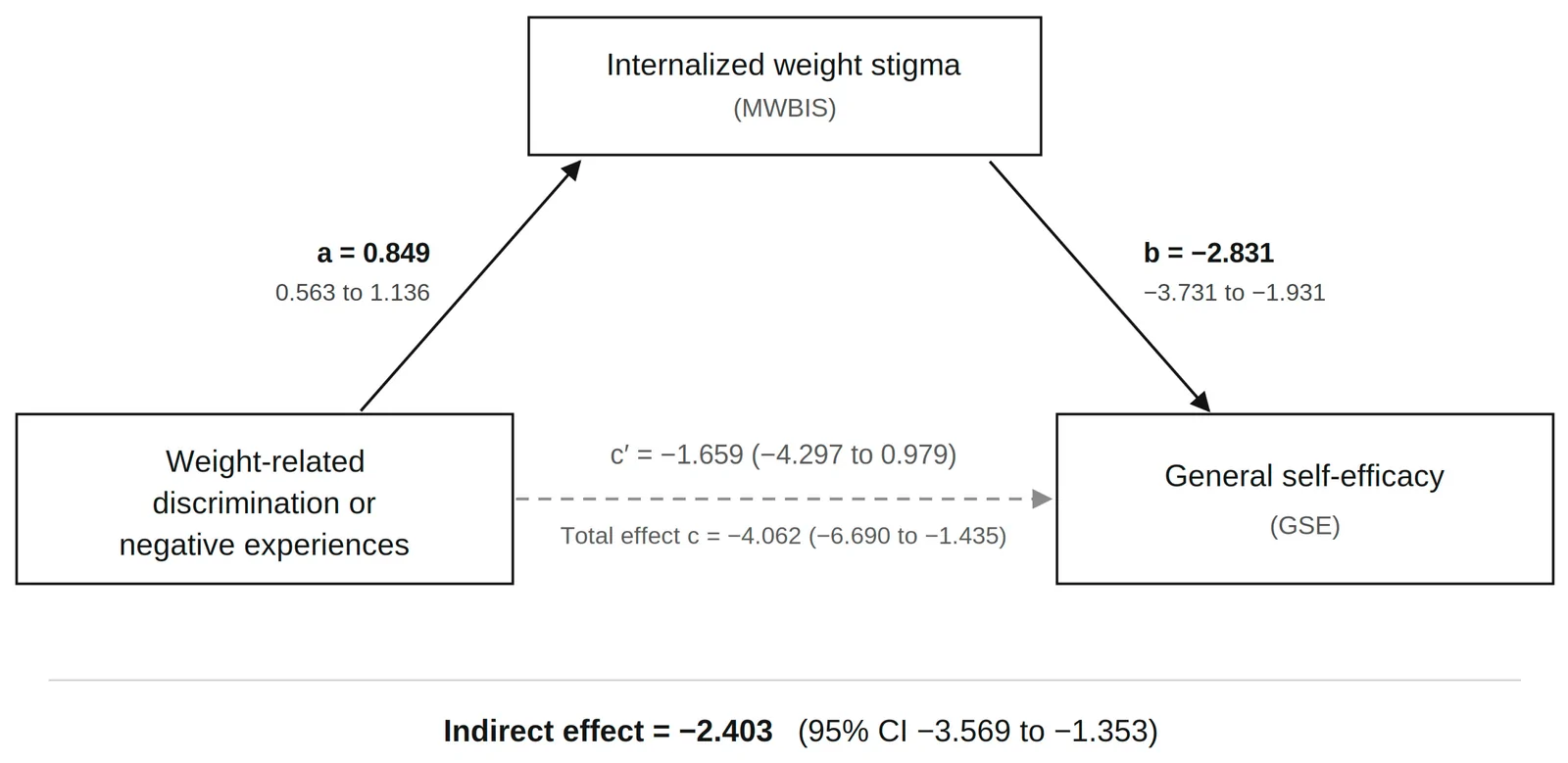
Indirect-effect model linking weight-related discrimination or negative experiences to general self-efficacy through internalized weight stigma. Unstandardized coefficients with 95% confidence intervals; *n* = 432. Adjusted for age, sex, and BMI; 5000 bootstrap resamples. The dashed line denotes a non-significant direct path (*p* = 0.217). MWBIS, Modified Weight Bias Internalization Scale; GSE, General Self-Efficacy Scale. Data are cross-sectional; arrows represent hypothesized associations and do not establish causal or temporal ordering.

A bootstrap-based indirect-effect analysis evaluated the hypothesized indirect association between weight-related discrimination or negative experiences and general self-efficacy through internalized weight stigma, adjusting for age, sex and BMI (Figure 2; Supplementary Table S3). Reporting such experiences was positively associated with internalized stigma (a path, *p* < 0.001), which was in turn negatively associated with general self-efficacy (b path, *p* < 0.001).

The total effect on self-efficacy was significant (B = −4.062, *p* = 0.003), whereas the direct effect was no longer significant once internalized stigma was entered (B = −1.659, *p* = 0.217); the indirect effect was significant (B = −2.403, 95% CI: −3.569 to −1.353). Sensitivity analysis using the full covariate set produced a similar indirect effect (B = −2.361, 95% CI: −3.586 to −1.328; *n* = 425).

### 3.5. Qualitative Findings

Four themes were identified: (1) Negotiating Obesity as a Label and Identity; (2) Living Under the Gaze: Judgment, Resistance, and Internalization; (3) When Weight Overshadows Care; and (4) Cycles of Weight Management and Conditional Confidence. Illustrative quotations are attributed using anonymized participant codes (K-1–K-20).

#### 3.5.1. Negotiating Obesity as a Label and Identity

Participants commonly distinguished the clinical meaning of obesity from the language they used to describe themselves in everyday life. “Overweight” or “having gained weight” was often preferred to “obese,” even among those who recognized that they met clinical criteria. For some, obesity became a meaningful self-description only when weight began to impair health or functioning: “Before 2017 I was obese too… but once it started to affect my health, it really became a disease—what you call obesity” (K-11). Others resisted the label altogether: “I would say, ‘I have gained some weight,’ but I would never say, ‘I am obese’” (K-16). Thus, obesity was negotiated not only as a BMI category but also through health, appearance, functioning, and self-definition.

#### 3.5.2. Living Under the Gaze: Judgment, Resistance, and Internalization

Weight-related judgment was described in family, social, occupational, and public settings. Comments about eating, appearance, willpower, relationships, and the need to lose weight were common, but their impact depended strongly on who delivered them. Remarks from parents, partners, and close relatives were often more influential than comments from strangers. Repeated criticism could be experienced as demotivating rather than encouraging: “They think saying these things will motivate me… but it does the opposite… Sometimes, even if I am going to exercise, I give up” (K-7).

Participants used different strategies to manage judgment, including ignoring comments, confronting others, avoiding particular settings, concealing their bodies, or making jokes about their own weight before others could do so. One participant described self-directed humour as a protective strategy: “Instead of letting them use it as a weapon, I used it as something in my own hand—my own card” (K-3). These responses did not necessarily mean that stigma was emotionally neutral. Some participants who initially described themselves as unaffected later reported sadness, reduced confidence, self-monitoring, or self-critical thoughts. Others explicitly applied negative stereotypes to themselves, including beliefs about lacking willpower. At the same time, several participants reported little or no direct discrimination, demonstrating variation in how external attitudes were experienced and internalized.

#### 3.5.3. When Weight Overshadows Care

Healthcare experiences ranged from supportive encounters to interactions perceived as dismissive or stigmatizing. Some participants described direct weight-related comments that caused embarrassment and subsequently influenced healthcare use. After a physician commented on her body during an examination, one participant recalled: “I felt very embarrassed. I never went to that doctor again; I made an appointment with someone else” (K-10).

Others described situations in which pain, psychological concerns, or other symptoms appeared to be overshadowed by weight, with consultations reduced to advice to “lose weight.” However, negative healthcare experiences were not universal. Several participants explicitly distinguished appropriate clinical advice from stigmatizing treatment and described respectful clinicians and the obesity center positively.

Structural barriers were also apparent. Standard-sized chairs, narrow toilet cubicles, imaging equipment, and fragmented movement between clinics generated concerns about fitting, breaking equipment, or completing care comfortably. One participant described worrying whether she would “fit inside” an MRI scanner or “break” an examination table or chair (K-8). These accounts indicated that healthcare difficulties could arise from both interpersonal interactions and the physical and organizational design of services.

#### 3.5.4. Cycles of Weight Management and Conditional Confidence

Participants described repeated weight-management attempts involving dietary changes, professional support, exercise, medication, or surgery. Weight loss was often followed by regain, while stress, emotional eating, health problems, sleep difficulties, and restrictive diets complicated maintenance. Confidence was therefore dynamic rather than stable: early success, supportive feedback, and improved functioning strengthened confidence, whereas regain, criticism, or perceived lapses undermined it.

For some participants, this loss of confidence extended beyond weight management into broader self-evaluation. After regaining weight following an initially successful attempt, one participant described thinking, “I probably cannot succeed at anything” (K-16). Others similarly described beginning an attempt with confidence but gradually losing motivation when change could not be sustained. Conversely, recent success and improvements in mobility or health could restore confidence. Future goals frequently emphasized health, mobility, breathing, and daily functioning, although appearance, clothing, and feeling comfortable in one’s body also remained important.

### 3.6. Integration of Quantitative and Qualitative Findings

Integration demonstrated both convergence and complementarity between the quantitative and qualitative findings. Weight-related discrimination or negative experiences were associated with greater internalized weight stigma and lower general self-efficacy, while qualitative accounts described shame, self-criticism, anticipated judgment, and withdrawal. Healthcare findings also converged: reports that negative weight-related encounters affected subsequent healthcare use were reflected in narratives of embarrassment, clinician avoidance, and weight overshadowing other health concerns. Qualitative findings expanded the quantitative results by identifying structural barriers and variation in responses to stigma. Finally, qualitative data complemented the indirect-effect findings by showing that weight-management confidence fluctuated with success, regain, criticism, and support. This context-dependent confidence was related to, but distinct from, domain-general self-efficacy. An overview of the integration of the quantitative and qualitative findings is presented in Table 4.

## 4. Discussion

This mixed-methods study found that self-reported weight-related discrimination or negative experiences in social or workplace settings were associated with greater internalized weight stigma and lower general self-efficacy among adults receiving obesity care. The hypothesized indirect association through internalized stigma was significant and robust to sensitivity analysis. Qualitative findings added context: external judgment was described as becoming self-criticism and demotivation, while repeated setbacks sometimes broadened into generalized doubts about competence. Healthcare findings differed: negative experiences were reported by a minority and were not independently associated with either psychometric outcome, yet most participants reporting them stated that subsequent healthcare use had been affected.

The distinction between experiencing stigma and internalizing it appears central. Meta-analytic evidence links greater weight-bias internalization to poorer psychosocial, physical, and behavioral health [6]. More directly, Bidstrup et al. reviewed 17 studies examining internalized weight stigma as an intervening variable between experienced/perceived stigma and biopsychosocial outcomes; evidence was strongest for disordered eating and preliminary for several other outcomes [7]. General self-efficacy was rarely examined. In a weight-management sample, Pearl et al. likewise found internalized weight bias to be more consistently associated than experienced stigma with adverse health-related correlates, including lower eating self-efficacy [26]. Our findings suggest that correlates of internalization may extend beyond weight-specific behaviors to broader beliefs about coping and competence.

The direction of association with self-efficacy requires caution. Hübner et al. modeled lower general self-efficacy as an antecedent of greater weight-bias internalization in bariatric surgery candidates [12], whereas Ramsamy et al. recently found greater internalized weight bias to be associated with lower obesity-specific self-efficacy, with coping fully accounting for that association [14]. Our model tested a different ordering and a domain-general outcome. Although the total association with GSE became non-significant after MWBIS was entered, the cross-sectional design cannot establish mediation, temporal sequence, or directionality; reciprocal processes remain plausible.

The modest explanatory power of the GSE model (R^2^ = 0.041) is also informative. General self-efficacy is broader than confidence in weight-management behaviors, and a weight-specific exposure would not be expected to explain most of its variance. The qualitative theme of conditional confidence helps interpret this distinction. Participants described confidence as changing with success, regain, criticism, support, and perceived lapses; for some, repeated failure extended into broader self-doubt. The qualitative data therefore complement the GSE result: weight-management confidence was context-dependent, whereas the scale captured a wider belief about coping and competence. Future studies should assess general and domain-specific self-efficacy concurrently and examine coping as a potential mechanism.

Higher BMI and female sex were independently associated with greater internalized stigma. Our finding of higher internalized weight stigma among women is consistent with a recent systematic review and meta-analysis reporting higher levels of internalized weight stigma in women than in men [27]. The BMI association may reflect greater exposure to weight-related judgment or greater salience of body-size differences, but these explanations cannot be distinguished here.

The qualitative findings also highlighted a distinction between clinical classification and self-identification. Although participants recognized that they met clinical criteria for obesity, several preferred everyday descriptions such as “overweight” or “having gained weight” rather than identifying themselves as “obese.” This suggests that clinically accurate terminology may not necessarily correspond to patients’ preferred self-descriptions. The finding reinforces the importance of patient-centered communication that separates clinical classification from personal identity and attends to individual preferences for weight-related terminology [28].

Healthcare experiences warrant separate interpretation. Weight-related negative healthcare experiences were reported by 11.5% of participants (50/434), whereas 88.5% did not report such experiences on this item. However, the absence of a reported negative experience should not be equated with satisfaction with care or the complete absence of weight stigma. By comparison, 27.8% of participants (120/432) reported weight-related discrimination or negative experiences in social or workplace settings. Although these findings suggest variation in reported experiences across settings, the two items assessed different domains and cannot be directly compared as equivalent measures of stigma. Importantly, 77.1% (37/48) of participants who reported negative healthcare experiences and answered the follow-up question indicated that these experiences had affected subsequent healthcare use. Multinational evidence links experienced and internalized weight stigma with healthcare avoidance, perceived judgment, and poorer care experiences [13], while a recent review similarly concluded that experienced, anticipated, and internalized stigma may reduce healthcare utilization and that structural barriers can restrict access [29]. Although the proportions reporting such experiences are not directly comparable, a 2025 mixed-methods study from a large academic medical center also documented delayed or cancelled care after weight-related treatment experiences, particularly among women with class III obesity [28]. Our qualitative accounts provide further context: participants described embarrassment, changing clinicians, weight overshadowing other symptoms, and unsuitable chairs, examination tables, toilets, and imaging equipment. Nevertheless, negative experiences were not universal, and some participants described respectful and supportive healthcare encounters, highlighting the heterogeneity of patients’ experiences. In line with this heterogeneity, over two-thirds of participants (68.7%, 299/435) considered the specialized obesity and diabetes center beneficial (Supplementary Results); this reflects perceived usefulness of the center rather than a direct measure of satisfaction with the care received. These findings are consistent with qualitative research documenting both stigmatizing and positive healthcare interactions [9] and with the interpersonal and systemic barriers synthesized by Ryan et al. [10]. They also align with a 2026 mixed-methods study identifying furniture, equipment, and access/infrastructure as core domains of obesity-inclusive hospital environments [30]. The relatively low proportion reporting weight-related negative healthcare experiences in our sample should be interpreted cautiously because exposure was measured with one binary item and questionnaires were administered face-to-face within the care setting. Beyond these measurement considerations, several explanations may account for the lower proportion of reported negative experiences in healthcare than in social or workplace settings. Supportive interactions within a specialized multidisciplinary obesity center may be one explanation, as some interview participants described respectful clinicians and positive experiences with the center. Differences in exposure across settings must also be considered: qualitative accounts of avoiding particular settings, concealing body size, and anticipating judgment suggest that some individuals may modify their social participation in ways that alter exposure to stigmatizing situations, and the relatively low proportion of employed participants (28.9%) indicates that opportunities for workplace-related experiences varied across the sample. These interpretations remain exploratory, as frequency of exposure to each setting, social participation, and avoidance behavior were not measured quantitatively; the lower reported frequency of negative healthcare experiences should therefore not be interpreted as evidence that weight stigma is less prevalent in healthcare settings.

These findings support stigma-informed obesity care addressing both internalization and the care environment. Reviews recommend education about the complex determinants of obesity, empathy-promoting and weight-inclusive approaches, and attention to structural barriers [31]. Persistent gaps in obesity education among newly qualified health professionals, particularly regarding patient-centered communication and weight stigma, underscore the need for such curricular reform [32]. A 2025 systematic review of 56 randomized trials found that interventions more consistently changed attitudinal outcomes than longer-term behavioral or clinical outcomes, underscoring the need for durable patient-centered evaluation [33]. In practice, respectful communication, privacy, size-inclusive furniture and equipment, accessible clinical spaces, and psychological support should complement efforts to reduce stigma at provider and institutional levels [30]. As Hill et al. emphasize, individual-level strategies must be embedded within broader structural approaches, encompassing institutional policies, physical environments, and system-level accountability [34]. This approach is compatible with current Turkish recommendations for multidisciplinary obesity care [35].

Beyond preventing stigmatizing encounters, health systems must also consider patients who have already experienced or internalized weight stigma. Acknowledging previous negative experiences, providing opportunities to voice concerns without judgment, ensuring continuity of care, establishing accessible feedback mechanisms supported by organizational accountability, and facilitating access to psychological support may help create conditions for rebuilding trust and re-engagement with care. Because evidence on the effectiveness of specific trust-restoration and re-engagement strategies remains limited, these are presented as practical implications requiring evaluation rather than as interventions demonstrated to be effective. Patient-informed evidence offers direction for such efforts: consumers of weight-management services have described peer support, health-coach support, and holistic, centralized, and flexible service formats as core components of care with which they would engage [36].

Moreover, because weight-related discrimination or negative experiences were reported considerably more often in social and workplace settings (27.8%) than in healthcare (11.5%), and contact with health services is episodic whereas social and occupational environments are everyday determinants involving repeated exposure to weight-related stereotypes and negative treatment, stigma reduction cannot be confined to healthcare. Potential strategies include workplace policies explicitly addressing weight-based harassment and discriminatory treatment, training for employers and employees, public education campaigns challenging assumptions that obesity is solely a matter of personal responsibility, and responsible media communication that avoids stigmatizing representations [17]. Meaningful involvement of people living with obesity in the development and evaluation of these initiatives is essential to ensure that interventions reflect their lived experiences and priorities; consistent with this, our participants most frequently endorsed structured support programs (72.0%), education (55.9%), and media campaigns (54.9%) as strategies for reducing weight stigma (Supplementary Results). International consensus recommendations likewise emphasize that reducing weight stigma requires coordinated action across healthcare, education, media, public health, and policy sectors, including measures to prevent weight-based discrimination and to improve the accessibility of healthcare environments [37]. Notably, a systematic review of obesity-related policies found that attributing obesity primarily to individual responsibility was associated with support for more punitive policies, whereas acknowledging broader environmental determinants was associated with more supportive policy attitudes, underscoring the importance of how obesity is framed in public communication [38]. Such approaches should complement healthcare-based interventions within broader, multilevel efforts to reduce weight stigma [17,31], and further research is needed to determine which strategies effectively reduce weight-related discrimination and improve psychosocial outcomes in everyday settings.

Future research should build on these qualitative findings by exploring how experiences of weight-related stigma and their internalization evolve over time and interact with both weight-management confidence and general self-efficacy. Longitudinal qualitative and mixed-methods studies could help clarify these processes and the circumstances under which stigmatizing experiences influence subsequent healthcare engagement. Particular attention should be given to individuals who delay or discontinue care following negative encounters, as their perspectives may be underrepresented in treatment-seeking samples. Participatory research involving people living with obesity could also inform the development and evaluation of strategies to rebuild trust, facilitate re-engagement with healthcare, and address stigma-related barriers at interpersonal and institutional levels.

### Strengths and Limitations

Strengths include consecutive recruitment from a specialized public obesity and diabetes center, validated Turkish measures, bootstrap-based indirect-effect analysis with sensitivity analysis, and integration of quantitative and qualitative findings from the same clinical population. The joint display made convergence and complementarity between the quantitative and qualitative findings explicit.

Several limitations should be considered. The cross-sectional design precludes causal or temporal inference, and reverse or bidirectional associations remain plausible. Social/workplace and healthcare experiences were each assessed with a single researcher-developed binary item without formal cognitive or pilot testing; frequency, severity, source, timing, and cumulative exposure were not captured. Face-to-face administration may have introduced social-desirability or courtesy bias, particularly for healthcare experiences, and recruitment from a treatment-seeking sample may underrepresent people who disengaged from services because of stigma. Relatedly, because the number and characteristics of eligible individuals who declined participation were not systematically recorded, participants could not be compared with non-participants, introducing the possibility of non-participation bias. Individuals who had experienced stigmatizing healthcare encounters or had disengaged from obesity care might have been less likely to participate, potentially leading to underrepresentation of negative experiences; conversely, individuals with particularly salient experiences of weight-related stigma might have been more motivated to participate. Such differences could have influenced both the reported frequency of negative experiences and the observed associations with internalized weight stigma and general self-efficacy, and the direction and magnitude of this potential bias cannot be determined from the available data. Height and weight were self-reported, and the single-center setting limits generalizability. Qualitative interviews followed questionnaire completion in the same population and therefore contextualize rather than independently validate the quantitative findings. Finally, qualitative accounts mainly concerned weight-management confidence, which is related to but distinct from domain-general self-efficacy, and the low GSE R^2^ indicates that important determinants were unmeasured. In addition, the total number of patients attending the center and the number assessed for eligibility were not systematically recorded, preventing calculation of a participation rate. For the qualitative phase, the absence of a formally documented saturation assessment and of a prospective sample-size rationale should be considered when interpreting the scope and transferability of the findings.

## 5. Conclusions

Weight-related negative experiences, internalized weight stigma, and lower general self-efficacy were interrelated in this treatment-seeking population, with a significant indirect association through internalized stigma. Qualitative accounts showed how external judgment could become self-critical evaluation while confidence fluctuated with context and recent outcomes. Healthcare-related negative experiences were not independently associated with either psychometric outcome, but when reported they were frequently accompanied by changes in subsequent care use and by interpersonal and structural barriers. Longitudinal, multisite studies using more detailed stigma measures and concurrent general and domain-specific self-efficacy assessments are needed to establish temporal ordering and identify modifiable mechanisms.

## Figures and Tables

**Table 1:** Sociodemographic Characteristics and Frequency of Self-Reported Weight-Related Discrimination or Negative Experiences.

Variable	Total Participants *n* (%)	Reported Discrimination/Negative Experiences *n*/N (%)	*p* Value
**Sex**			0.008
Female	322 (74.0)	100/321 (31.2)	
Male	113 (26.0)	20/111 (18.0)	
**Marital status**			0.009
Single	84 (19.3)	34/84 (40.5)	
Married	321 (73.8)	81/318 (25.5)	
Divorced/Widowed	30 (6.9)	5/30 (16.7)	
**Education level**			0.023
No formal education	18 (4.1)	3/18 (16.7)	
Primary school	194 (44.6)	41/193 (21.2)	
High school	111 (25.5)	36/110 (32.7)	
University	99 (22.8)	34/98 (34.7)	
Postgraduate	13 (3.0)	6/13 (46.2)	
**Employment status**			<0.001
Employed	125 (28.9)	35/123 (28.5)	
Unemployed	216 (49.9)	56/216 (25.9)	
Retired	65 (15.0)	9/64 (14.1)	
Student	27 (6.2)	18/27 (66.7)	
**Obesity class**			<0.001
Class I (30.0–34.9)	175 (40.2)	29/173 (16.8)	
Class II (35.0–39.9)	135 (31.0)	31/135 (23.0)	
Class III (≥40.0)	125 (28.7)	60/124 (48.4)	
**Perceived income level**			0.179
Very low	60 (14.0)	21/59 (35.6)	
Low	155 (36.1)	34/155 (21.9)	
Middle	200 (46.6)	58/198 (29.3)	
High	14 (3.3)	4/14 (28.6)	

Note: Percentages in the third column are based on valid responses to the discrimination item and may therefore use denominators smaller than the subgroup totals shown in the second column; missing values were excluded without imputation. Pearson χ^2^ tests were used, except for education level and perceived income level, for which the Fisher–Freeman–Halton exact test was used. Age and BMI are reported in the text. Bold text indicates category headings for the variables presented in the table.

**Table 2:** MWBIS and GSE Scores According to Self-Reported Weight-Related Experiences.

Variable	MWBIS Median (IQR)	*p *†	GSE Median (IQR)	*p* †
**Weight-related discrimination or negative experiences**		**<0.001**		**<0.001**
Yes (*n* = 120)	4.23 (1.73)		61.00 (14.75)	
No (*n* = 312)	3.00 (1.91)		67.00 (16.00)	
**Weight-related negative experiences in healthcare**		**0.043**		0.726
Yes (*n* = 50)	3.86 (2.02)		64.50 (18.25)	
No (*n* = 384)	3.36 (2.18)		65.00 (16.00)	

MWBIS, Modified Weight Bias Internalization Scale; GSE, General Self-Efficacy Scale; IQR, interquartile range. Note: † Mann–Whitney U test. Bold indicates *p* < 0.05.

**Table 3:** Multivariable Regression Models for Internalized Weight Stigma and Self-Efficacy.

Variable	MWBIS—B (95% CI)	*p* Value	GSE—B (95% CI)	*p* Value
BMI	0.031 (0.011 to 0.052)	0.003	−0.064 (−0.254 to 0.126)	0.511
Age	−0.004 (−0.012 to 0.005)	0.373	0.032 (−0.046 to 0.110)	0.418
**Sex (ref: male)**				
Female	0.425 (0.146 to 0.704)	0.003	−1.472 (−4.067 to 1.123)	0.265
**Income level (ref: middle)**				
Very low/low income	0.162 (−0.085 to 0.410)	0.198	0.210 (−2.092 to 2.512)	0.858
High income	−0.070 (−0.751 to 0.611)	0.840	0.886 (−5.445 to 7.217)	0.783
**Weight-related discrimination/negative experiences (ref: no)**				
Yes	0.814 (0.525 to 1.103)	<0.001	−4.238 (−6.925 to −1.551)	0.002
**Weight-related negative experiences in healthcare (ref: no)**				
Yes	0.192 (−0.187 to 0.571)	0.319	0.946 (−2.575 to 4.466)	0.598

Note: Unstandardized regression coefficients (B) with 95% confidence intervals are presented. Reference categories are shown in the table. All variables were entered simultaneously into the multivariable model. Both models used complete cases and were based on *n* = 425 participants (97.7% of the sample); ten participants with missing data on at least one model variable were excluded. MWBIS model: F(7, 417) = 12.349, *p* < 0.001, R^2^ = 0.172, adjusted R^2^ = 0.158. GSE model: F(7, 417) = 2.570, *p* = 0.013, R^2^ = 0.041, adjusted R^2^ = 0.025. Bold text indicates category headings for the variables presented in the table.

**Table 4:** Joint Display of Quantitative and Qualitative Findings.

Quantitative Finding	Corresponding Qualitative Theme	Representative Quotation	Integrated Interpretation
Weight-related discrimination or negative experiences were independently associated with higher internalized weight stigma (B = 0.814, 95% CI 0.525 to 1.103; *p* < 0.001) and were associated with lower general self-efficacy.	Living Under the Gaze: Judgment, Resistance, and Internalization	“They think saying these things will motivate me… but it does the opposite… Sometimes, even if I am going to exercise, I give up.” (K-7)	External judgment was described as contributing to self-critical evaluation and demotivation, providing experiential context for the observed quantitative associations. Convergence.
Among participants reporting weight-related negative experiences in healthcare, 77.1% (37/48) reported that subsequent healthcare use was affected.	When Weight Overshadows Care	“I felt very embarrassed. I never went to that doctor again; I made an appointment with someone else.” (K-10)	Qualitative accounts showed how a stigmatizing encounter could influence subsequent care-seeking and choice of clinician. Convergence.
The indirect association between weight-related discrimination or negative experiences and general self-efficacy through internalized weight stigma was significant (B = −2.403, 95% CI −3.569 to −1.353).	Living Under the Gaze; Cycles of Weight Management and Conditional Confidence	“Then I get demoralized and think, ‘I probably cannot succeed at anything.’” (K-16)	Some accounts described self-doubt extending beyond weight management, consistent with—but not establishing—the hypothesized indirect association. Convergence with complementarity.
The multivariable model explained a limited proportion of variance in general self-efficacy (R^2^ = 0.041; adjusted R^2^ = 0.025).	Cycles of Weight Management and Conditional Confidence	“I felt confident at the beginning, but over time that confidence decreases.” (K-1)	Weight-management confidence was described as dynamic and context-dependent, whereas the quantitative GSE measure captured broader domain-general self-efficacy. The qualitative findings therefore expanded rather than duplicated the quantitative result. Complementarity and expansion.

Note: MWBIS, Modified Weight Bias Internalization Scale; GSE, General Self-Efficacy Scale. Illustrative quotations are attributed using anonymized participant codes (K-1–K-20). Integrated interpretations are based on convergence, complementarity, or expansion across the two datasets. Because the data are cross-sectional, these interpretations do not establish causal or temporal ordering.

## Data Availability

The data presented in this study are available on request from the corresponding author due to privacy and ethical restrictions.

## Supplementary Materials

The following supporting information can be downloaded at: https://www.mdpi.com/article/10.3390/healthcare14203510/s1. Supplementary Material S1: Structured questionnaire; Supplementary Material S2: Semi-structured interview guide; Supplementary Material S3: STROBE checklist; Supplementary Material S4: COREQ checklist; Table S1: MWBIS and GSE Scores Across Sociodemographic Characteristics and Obesity Class; Table S2: Correlation Matrix between MWBIS, GSE, BMI, Age, and Household Monthly Income; Table S3: Indirect-Effect Analysis of the Association Between Weight-Related Discrimination or Negative Experiences and General Self-Efficacy via Internalized Weight Stigma; Table S4: Characteristics of Participants in the Qualitative Phase.
