# Supplementary material for: Weight-Related Negative Experiences, Internalized Weight Stigma, and General Self-Efficacy in Adults with Obesity: An Explanatory Sequential Mixed-Methods Study
Source: Healthcare (Basel). 2026 Oct 19;14(20):3510. doi: 10.3390/healthcare14203510 (PMC13704752; doi:10.3390/healthcare14203510)
Supplement: Supplementary file 1 [file healthcare-14-03510-s001.zip › Supplementary_Material_S1_Structured_Questionnaire.pdf]

Weight-Related Negative Experiences, Internalized Weight Stigma, and General Self-Efficacy in Adults with Obesity: An Explanatory Sequential Mixed-Methods Study

SUPPLEMENTARY MATERIAL S1. Full Structured Questionnaire

Dear Participant,

This questionnaire has been prepared for a study conducted by the Department of Public Health, Faculty of Medicine, Zonguldak Bülent Ecevit University. The data collected will be used solely for the purposes of this study. Participation is voluntary. Thank you for your contribution.

1. Please indicate your height and body weight.

   Height (cm): _______ *Weight (kg):* ________

2. What is your age? _______

3. What is your gender?

   a. Female

   b. Male

4. What is your highest level of education?

   a. No formal education

   b. Primary school

   c. Secondary school

   d. High school

   e. University

   f. Master's–Doctorate

5. What is your marital status?

   a. Single

   b. Married

c. Divorced

d. Widowed

6. Place of residence?

a. Village

b. District center

c. City center

7. What is your employment status?

a. Employed

b. Unemployed

c. Homemaker

d. Retired

e. Student

8. What is the total monthly household income? _______

9. How would you rate your income level?

a. Very low

b. Low

c. Middle

d. High

e. Very high

10. What is the TOTAL number of people living in your household? _______

11. How many people in your household are employed? _______

12. Do you have health insurance?

a. None

b. State-covered (SGK)

c. Private insurance

d. Private + State insurance

13. Have you ever consulted a specialist for weight management?

a. Yes

b. No

14. Do you have a physician-diagnosed chronic disease?

a. Yes

b. No

15. If yes, which of the following do you have? (You may select more than one option)

a. Diabetes

b. Hypertension

c. Cardiovascular disease

d. Thyroid disorders

e. Asthma or COPD

f. Mental disorders (such as depression or anxiety)

g. Kidney diseases

h. Musculoskeletal disorders (e.g., arthritis)

i. Gastrointestinal disorders (e.g., reflux, irritable bowel syndrome)

j. Other: ___________

16. Do you think your weight affects your work life?

a. Does not affect at all

b. Affects slightly

c. Affects moderately

d. Affects greatly

17. Have you ever experienced discrimination or a negative experience at work or in society because of your weight?

a. Yes

b. No

18. Do you think your experience of stigma affects your social life?

a. Not affected at all

b. Slightly affected

c. Moderately affected

d. Greatly affected

19. What kinds of perceptions or attitudes toward overweight individuals do you encounter in society and your surroundings? (You may select more than one option)

a. Overweight individuals are perceived as lacking willpower.

b. Overweight individuals are perceived as having an unhealthy lifestyle.

c. Overweight individuals are perceived as having limited social lives.

d. Overweight individuals are perceived as emotionally weak.

e. People tend to feel sorry for overweight individuals due to their health problems.

f. My community and social environment support overweight individuals in their social lives.

g. Mocking or derogatory behaviors toward overweight individuals are observed.

h. There is a tendency to question the achievements of overweight individuals.

i. Other: ______________

20. In which of the following situations have you felt uncomfortable or inadequate because of your weight? (You may select more than one option)

a. At the workplace or during job interviews

b. Within the family

c. In friendships

d. In romantic relationships

e. When participating in social activities

f. When using public transportation

g. In restaurants or cafés

h. In gyms

i. While shopping

j. When accessing healthcare services

k. None

21. In which of the following situations do you think you have been exposed to disturbing behavior or negative attitudes because of your weight? (You may select more than one option)

a. At the workplace or during job interviews

b. Within the family

c. In friendships

d. In romantic relationships

e. When participating in social activities

f. When using public transportation

g. In restaurants or cafés

h. In gyms

i. While shopping

j. When accessing healthcare services

k. None

22. What effects do societal perceptions toward overweight individuals have on you? (You may select more than one option)

a. It makes me feel insecure in society.

b. It negatively affects my self-esteem.

c. It harms my social relationships.

d. It causes me to hesitate in seeking healthcare.

e. It has no effect.

f. Other: _____________

23. Have you received any psychological support because of your weight?

a. Yes

b. No

24. Have you experienced any discrimination or negative attitudes related to weight stigma while receiving healthcare services?

a. Yes

b. No

25. How have the attitudes of healthcare professionals toward your weight affected your experience of accessing healthcare services?

a. Not affected at all

b. Slightly affected

c. Moderately affected

d. Greatly affected

26. Weight stigma refers to negative attitudes or discrimination individuals face due to their weight. Which of the following practices do you think should be implemented to reduce weight stigma? (You may select more than one option)

a. Organizing public awareness campaigns

b. Implementing regulations in the media to reduce negative perceptions related to weight

c. Adding educational content to curricula to raise awareness about weight stigma

d. Developing special support programs for individuals with obesity (psychological counseling, group therapy, etc.)

e. Other: _____________

27. Weight stigma refers to negative attitudes or discrimination individuals face due to their weight. What changes do you think should be made in healthcare services to prevent weight stigma? (You may select more than one option)

a. Developing patient-friendly procedures regarding weight stigma in healthcare services

b. Expanding specialized clinical services for individuals with obesity

c. Allocating more specialists and resources for weight management in healthcare

d. Establishing psychological support units in obesity centers

e. Providing regular training for healthcare professionals on weight stigma

f. Activating patient feedback mechanisms (e.g., surveys, complaint systems)

g. Other: _____________

28. Do you think clinics such as Obesity and Diabetes Centers are beneficial for obesity management and meeting individual needs? If you have any suggestions, please specify.

a. Yes, very beneficial

b. Yes, but there are some shortcomings

c. No, not sufficiently beneficial

d. No, I find them ineffective

e. Other: _____________
