# Supplementary material for: Weight-Related Negative Experiences, Internalized Weight Stigma, and General Self-Efficacy in Adults with Obesity: An Explanatory Sequential Mixed-Methods Study
Source: Healthcare (Basel). 2026 Oct 19;14(20):3510. doi: 10.3390/healthcare14203510 (PMC13704752; doi:10.3390/healthcare14203510)
Supplement: Supplementary file 1 [file healthcare-14-03510-s001.zip › Supplementary_Material_S2_Semi-Structured_Interview_Guide.pdf]

Weight-Related Negative Experiences, Internalized Weight Stigma, and General Self-Efficacy in Adults with Obesity: An Explanatory Sequential Mixed-Methods Study

SUPPLEMENTARY MATERIAL S2. Semi-Structured Interview Guide

Qualitative Interview Questions

SECTION 1 – General Perception and Life Experience

1. How do you define obesity? For you, does obesity represent a health problem, an aesthetic concern, or another condition?
2. How do you think obesity and body weight are perceived in society? Do you feel that this perception creates any pressure on you?
3. What has been the most impactful event for you emotionally, socially, or professionally related to your weight? Could you describe this experience?
4. Have you experienced any difficulties in your daily life, social environment, workplace, or family due to your weight? If yes, could you describe them?

SECTION 2 – Healthcare Experience and Stigma

5. Have you ever felt that you experienced stigma, discrimination, or prejudice related to your weight in a healthcare setting? If yes, could you describe this experience?
6. Have there been situations in which you felt that your weight influenced the way healthcare professionals (physicians, nurses, dietitians, etc.) approached you? How did you evaluate this approach?
7. During healthcare services, did you feel that the examinations, treatments, or explanations provided to you were appropriate for your needs? Did you encounter delays, neglect, or blaming attitudes?

SECTION 3 – Self-Efficacy and Weight Management

8. Have you ever attempted weight management, weight loss, or adopting a healthy lifestyle? If yes, could you describe this process and your motivation?
9. Do you currently have a goal related to managing your weight or losing weight? Is this goal a health necessity or a personal preference?
10. How confident do you feel about managing your weight? Has this confidence increased or decreased over time?
11. What were the biggest challenges you faced during the weight management process? How did you cope with these challenges, and were you able to receive support from your environment?

SECTION 4 – Psychosocial Effects and Internalization

12. What are the attitudes of people around you regarding your weight? How have these attitudes affected your social relationships (friendships, family relationships, romantic relationships)?
13. Do you experience negative self-talk, self-blame, or thoughts that affect your self-perception related to your weight? In which situations do these thoughts increase?
14. How do these negative thoughts influence your behaviors (e.g., anger, withdrawal, self-restriction, eating behaviors, etc.), and how do you cope with them?

SECTION 5 – Health Policies and Recommendations

15. In your opinion, what changes should be made within the healthcare system and healthcare institutions to ensure better services for individuals with excess weight? Additionally, what are your suggestions regarding the obesity center where you receive services?
