# Supplementary material for: Weight-Related Negative Experiences, Internalized Weight Stigma, and General Self-Efficacy in Adults with Obesity: An Explanatory Sequential Mixed-Methods Study
Source: Healthcare (Basel). 2026 Oct 19;14(20):3510. doi: 10.3390/healthcare14203510 (PMC13704752; doi:10.3390/healthcare14203510)
Supplement: Supplementary file 1 [file healthcare-14-03510-s001.zip › Supplementary_Material_S4_COREQ_Checklist.pdf]

**COREQ 32-ITEM CHECKLIST**
**for**

**Weight-Related Negative Experiences, Internalized Weight Stigma, and General Self-Efficacy in Adults with Obesity: An Explanatory Sequential Mixed-Methods Study**

| Item | Guide questions/description | Section |
|---|---|---|
| Domain 1: Research team and reflexivity | | |
| 1. Interviewer/facilitator | Which author/s conducted the interviews or FGDs? | Methods (Qualitative Data Collection and Analysis) |
| 2. Credentials | What were/are the researcher’s credentials? | Methods (Qualitative Data Collection and Analysis) |
| 3. Occupation | What was their occupation at the time of the study? | Methods (Qualitative Data Collection and Analysis) |
| 4. Gender | Was the researcher male or female? | Methods (Qualitative Data Collection and Analysis) |
| 5. Experience and training | What experience or training did the researcher have? | Methods (Qualitative Data Collection and Analysis) |
| 6. Relationship with participants established | Was a relationship with participants established prior to study commencement? | Methods (Qualitative Data Collection and Analysis) |
| 7. Participant knowledge of the interviewer | What did the participants know about the researcher? | Methods (Qualitative Data Collection and Analysis) |
| 8. Interviewer characteristics | What characteristics were reported about the interviewer/facilitator? | Methods (Qualitative Data Collection and Analysis) |
| Domain 2: study design | | |
| 9. Methodological orientation and Theory | What methodological orientation was stated to underpin the study? | Methods – Study Design and Setting; Methods – Qualitative Data Collection and Analysis (thematic analysis) |
| 10. Sampling | How were participants or articles selected? | Methods – Participants and Eligibility Criteria (qualitative |
| 11. Method of approach | How were participants approached? | Methods – Participants and Eligibility Criteria (qualitative |
| 12. Sample size | How many participants or articles were in the study? | Methods – Participants and Eligibility Criteria (n=20) |

| | | |
|---|---|---|
| 13. Non-participation | How many people refused to participate or dropped out, or articles were excluded during review? For what reasons? | Methods – Participants and Eligibility Criteria (not systematically recorded); Discussion – Strengths and Limitations |
| 14. Setting of data collection | Where was the data collected or what regions of articles are represented? | Methods – Qualitative Data Collection and Analysis |
| 15. Presence of non-participants | Was anyone else present besides the participants and researchers? | Methods (Qualitative Data Collection and Analysis) |
| 16. Description of sample | What are the important characteristics of the sample? | Results – Participant Characteristics; Supplementary Table S4 |
| 17. Interview guide or alternative data collection tool | Were examples from questions, prompts, guides provided by the authors? | Methods – Qualitative Data Collection and Analysis; Supplementary Material 2 |
| 18. Repeat interviews | Were repeat interviews carried out, or were multiple articles from the same study reviewed? | No; each participant was interviewed once |
| 19. Audio/visual recording | Did the research use audio or visual recording to collect the data? | Methods – Qualitative Data Collection and Analysis |
| 20. Field notes | Were field notes made during and/or after the interview/FGD or article review? | No formal field notes were made |
| 21. Duration/Length | What was the duration of the interviews/FGDs or length of the articles? | Methods (Qualitative Data Collection and Analysis) |
| 22. Data saturation | Was data saturation discussed? | Methods – Participants and Eligibility Criteria (qualitative phase); Methods – Study Size |
| 23. Transcripts returned | Were transcripts returned to participants for comment and/or correction? | No; transcripts were not returned to participants |
| **Domain 3: analysis and findings** | | |
| 24. Number of data coders | How many coders helped code the data? | Methods – Qualitative Data Collection and Analysis (two |
| 25. Description of the coding tree | Did authors provide a description of the coding tree (ie: codebook)? | Methods – Qualitative Data Collection and Analysis (codes grouped into subthemes and |

| | | |
|---|---|---|
| 26. Derivation of themes | Were codes/categories identified in advance (deductive) or derived from the data (inductive)? | Methods – Qualitative Data Collection and Analysis |
| 27. Software | What software, if applicable, was used to manage the data? | Methods – Qualitative Data Collection and Analysis (MAXQDA Analytics Pro 2024) |
| 28. Participant checking | Did, or will, participants or article authors provide feedback on the findings/results? | No; participants did not provide feedback on the findings |
| 29. Quotations presented | Were participant quotations or article text presented to illustrate the themes/findings/results? Was each quotation identified? | Results |
| 30. Data and findings consistent | Was there consistency between the data presented and the identified themes? | Results |
| 31. Clarity of major themes | Were major themes clearly presented in the findings? | Results |
| 32. Clarity of minor themes | Is there a description of diverse cases or discussion of minor themes? | Results |
