# Supplementary material for: Weight-Related Negative Experiences, Internalized Weight Stigma, and General Self-Efficacy in Adults with Obesity: An Explanatory Sequential Mixed-Methods Study
Source: Healthcare (Basel). 2026 Oct 19;14(20):3510. doi: 10.3390/healthcare14203510 (PMC13704752; doi:10.3390/healthcare14203510)
Supplement: Supplementary file 1 [file healthcare-14-03510-s001.zip › Supplementary_Tables_S1_S4.pdf]

Weight-Related Negative Experiences, Internalized Weight Stigma, and General Self-Efficacy in Adults with Obesity: An Explanatory Sequential Mixed-Methods Study

SUPPLEMENTARY TABLES S1–S4

Supplementary Results

Weight-Management and Psychological Support

Professional and psychological weight-management support were reported by 58.1% and 8.5% of participants, respectively. Psychological support was more common among individuals reporting weight-related discrimination or negative experiences (15.8%, p=.001). Both forms of support were associated with higher MWBIS and lower GSE scores (professional support: MWBIS 3.68 vs. 3.00, p<.001; GSE 63.50 vs. 68.00, p=.004; psychological support: MWBIS 4.18 vs. 3.36, p=.002; GSE 58.00 vs. 65.00, p=.026). Because all measures were concurrent, these associations may equally reflect greater distress prompting help-seeking.

Participant Perspectives on Obesity Care

All 435 participants responded to these items. The Obesity and Diabetes Center was considered beneficial by 68.7% (299/435), whereas 22.5% (98/435) reported that it had shortcomings. Expanding structured support programs was the most frequently endorsed strategy for reducing weight stigma (72.0%, 313/435), followed by education (55.9%, 243/435) and media campaigns (54.9%, 239/435).

Supplementary Table S1. MWBIS and GSE Scores Across Sociodemographic Characteristics and Obesity Class

Variable	MWBIS median (IQR)	p	GSE median (IQR)	p
**Sex**		**<.001 †**		**.036 †**
Female	3.64 (2.11)		64.00 (16.25)	
Male	2.91 (2.09)		68.00 (14.50)	
**Marital status**		.800 ‡		**.003 ‡**
Single	3.73 (2.23)		60.00 (15.75)	
Married	3.27 (2.14)		65.00 (16.00)	
Divorced/Widowed	3.14 (2.20)		65.00 (20.50)	
**Education level**		.584 ‡		.502 ‡
No formal education	3.86 (3.14)		63.00 (15.50)	
Primary school	3.45 (2.02)		66.00 (17.00)	
High school	3.64 (2.36)		64.00 (15.00)	

Variable	MWBIS median (IQR)	p	GSE median (IQR)	p
University	3.27 (1.91)		65.00 (17.00)	
Postgraduate	3.00 (2.14)		64.00 (18.00)	
**Employment status**		**<.001 ‡**		**.032 ‡**
Employed	3.36 (2.14)		65.00 (15.00)	
Unemployed	3.45 (2.07)		65.00 (16.00)	
Retired	2.91 (1.77)		68.00 (18.00)	
Student	5.09 (2.09)		60.00 (14.00)	
**Perceived income level**		.536 ‡		.756 ‡
Very low	3.27 (2.61)		63.00 (16.75)	
Low	3.45 (2.09)		66.00 (17.00)	
Middle	3.36 (2.07)		65.00 (15.00)	
High	3.18 (2.02)		66.00 (16.50)	
**Obesity class**		**<.001 ‡**		.079 ‡
Class I (30.0–34.9)	3.00 (2.18)		68.00 (16.00)	
Class II (35.0–39.9)	3.45 (2.09)		65.00 (18.00)	
Class III (≥40.0)	3.91 (1.95)		63.00 (13.00)	

MWBIS, Modified Weight Bias Internalization Scale; GSE, General Self-Efficacy Scale; IQR, interquartile range. Note: † Mann-Whitney U test. ‡ Kruskal-Wallis test; Dunn-Bonferroni post hoc comparisons were performed following significant omnibus tests. For MWBIS across obesity classes, Class III differed from Class I (p<.001) and Class II (p=.027), whereas Class I and Class II did not differ (p=.098). Bold indicates p<.05. Analyses were based on available data for each variable (n=430–435).

Supplementary Table S2. Correlation Matrix between MWBIS, GSE, BMI, Age, and Household Monthly Income

Variable	MWBIS	GSE	BMI	Age	Household income
MWBIS	1				
GSE	-.35***	1			
BMI	.26***	-.12*	1		
Age	-.12*	.09	-.09	1	
Household monthly income	-.11*	.11*	-.13**	-.32***	1

Note: Values are Spearman rank correlation coefficients (ρ). Household monthly income is the reported total monthly household income in Turkish lira and was available for 410 participants; all other coefficients are based on n=435. This variable is distinct from the

categorical perceived income level used in the multivariable models (Table 3). *p<.05, **p<.01, ***p<.001.

Supplementary Table S3. Indirect-Effect Analysis of the Association Between Weight-Related Discrimination or Negative Experiences and General Self-Efficacy via Internalized Weight Stigma

Effect type	B	95% CI	p value
Discrimination/negative experiences → Internalized weight stigma (a path)	0.849	0.563 to 1.136	<.001
Internalized weight stigma → GSE (b path)	-2.831	-3.731 to -1.931	<.001
Total effect (c path)	-4.062	-6.690 to -1.435	.003
Direct effect (c’ path)	-1.659	-4.297 to 0.979	.217
Indirect effect (a × b)	-2.403	-3.569 to -1.353	—

Note: Unstandardized coefficients (B) are presented. Indirect effects were tested using bootstrap confidence intervals based on 5,000 resamples. Models were adjusted for age, sex, and BMI, and were based on n=432 participants with complete data on all model variables; the sensitivity analysis using the full covariate set was based on n=425. An indirect effect was considered statistically significant when the 95% bootstrap confidence interval did not include zero.

Supplementary Table S4. Characteristics of Participants in the Qualitative Phase

Participant code	Age (years)	Sex	Obesity class	MWBIS score	GSE score
K-1	27	Female	III	4.18	48
K-2	26	Female	III	5.64	36
K-3	31	Female	II	4.82	64
K-4	21	Male	III	2.82	68
K-5	50	Female	I	3.82	60
K-6	44	Female	II	5.73	41
K-7	29	Female	I	2.82	66
K-8	23	Male	III	2.36	56
K-9	50	Female	III	3.09	57
K-10	40	Female	III	5.09	53
K-11	30	Male	III	4.55	48
K-12	37	Male	III	4.45	44
K-13	31	Female	III	3.82	64
K-14	52	Female	I	1.36	69
K-15	50	Female	II	3.64	67

Participant code	Age (years)	Sex	Obesity class	MWBIS score	GSE score
K-16	43	Female	II	4.18	56
K-17	44	Female	III	5.09	64
K-18	24	Female	III	3.91	65
K-19	29	Female	I	4.36	49
K-20	29	Male	I	2.45	74

MWBIS, Modified Weight Bias Internalization Scale; GSE, General Self-Efficacy Scale. Obesity class was defined as Class I (30.0–34.9 kg/m^2), Class II (35.0–39.9 kg/m^2) or Class III (≥40.0 kg/m^2). MWBIS scores are means of 11 items (range 1–7) and GSE scores are totals of 17 items (range 17–85). All values are taken from the quantitative dataset. The qualitative sample comprised 15 women and 5 men, spanned all three obesity classes (Class I, n=5; Class II, n=4; Class III, n=11), and covered MWBIS scores from 1.36 to 5.73 and GSE scores from 36 to 74, spanning the lower and upper portions of the distributions observed in the quantitative sample.
